# Effect of Agar/AgNP Composite Film Packaging on Refrigerated Beef Loin Quality

**DOI:** 10.3390/membranes11100750

**Published:** 2021-09-29

**Authors:** Seok-In Hong, Youngjin Cho, Jong-Whan Rhim

**Affiliations:** 1 Korea Food Research Institute, 245 Nongsaengmyeong-ro, Iseo-myeon, Wanju-gun, Jeollabuk-do 55365, Korea; yjcho74@kfri.re.kr; 2 BioNanocomposite Research Center, Department of Food and Nutrition, Kyung Hee University, 26 Kyungheedae-ro, Dongdaemun-gu, Seoul 02447, Korea; jwrhim@khu.ac.kr

**Keywords:** agar, silver nanoparticles, composite film, beef loin, active packaging

## Abstract

Fresh beef loin was packaged with 0–2% silver nanoparticles (AgNPs) incorporated agar films to investigate the effect of antimicrobial packaging on meat quality changes in terms of microbiological and physicochemical properties. Raw beef cuts were directly inoculated with *Listeria monocytogenes* and *Escherichia coli* O157:H7 and stored in the air-sealed packages combined with the agar films at 5 °C for 15 days. Beef samples showed low susceptibility to the agar/AgNP composite films, resulting in about one log reduction of the inoculated pathogenic bacteria in viable cell count during storage. However, the composite films could partly prevent beef samples from directly contacting oxygen, maintaining the meat color and retarding oxidative rancidity. Experimental results suggested that the AgNP-incorporated agar films can potentially be applied in packaged raw meats as an active food packaging material to inhibit microbial and physicochemical quality deterioration during distribution and sale.

## 1. Introduction

Because meat has a high water activity and is rich in nutrients, it is highly susceptible to microbial contamination and growth, resulting in off-flavors and off-tastes, changes in texture, and slime formation. Additionally, rancidity and discoloration of meat are mainly caused by biochemical reactions such as lipid and myoglobin oxidation. The number of microorganisms on the surface of fresh meat increases during storage and distribution according to typical microbial growth patterns in response to temperature, pH, and oxygen availability. The degree and nature of surface contamination of cut meat are known to determine its potential shelf life [1].

Microbial growth in raw meat can cause not only poor quality but sometimes the development of foodborne diseases. Therefore, several techniques have been used to inhibit growth and retard bacterial activity, including temperature control, vacuum or modified atmospheric packaging, active packaging, lactic acid bacteria (LAB) application, organic acids, antioxidants, irradiation, and hot water [1,2,3,4,5,6,7,8]. Antimicrobial packaging has also shown beneficial effects in inhibiting spoilage and pathogenic microorganisms of concern in meat to maintain freshness, extend shelf-life and ensure food safety [9,10,11,12]. The effectiveness of antimicrobial packaging is usually assessed by measuring changes in the number of microorganisms or quality factors related to microbial growth [13].

Biopolymers produced from various natural resources such as polysaccharides, proteins, and their derivatives from plant and animal origins have been considered as attractive packaging materials because the use of biopolymers is a sustainable development approach that aids in environmental preservation [14,15]. Moreover, biopolymer-based packaging materials have beneficial properties in maintaining the quality of packaged foods and extending their shelf-life [16]. Biopolymers are also suitable for synthesizing and stabilizing silver nanoparticles (AgNPs), mainly providing a uniform dispersion of nanoparticles and structural integrity within the resulting nanocomposites. Silver nanoparticles have many applications in packaging and medical fields because of their unique properties, such as large specific surface area and strong antimicrobial activity with high stability [17]. Due to the potent antimicrobial properties of the biopolymer-based films containing AgNPs, they can be applied to various types of food packaging to prevent microbial growth and extend the shelf-life of food products [18,19]. In addition to their potent and broad-spectrum antimicrobial activity, the benefits of using AgNPs in biopolymer packaging are associated with few adverse effects on the sensory properties of foods and the potential for increased consumer acceptance [20].

Application tests of biopolymers, mainly as edible coatings, incorporated with AgNPs, have already been conducted on several foods such as fruit and vegetables, meats, and dairy products [20,21,22,23,24,25]. Ag-chitosan nanocomposites incorporated as edible coatings could extend the shelf-life of fresh-cut melon [25]. Coated samples with Ag-chitosan nanocomposites induced a microbial reduction of 0.6 Log units from days 10 to 13 at 5 °C. They showed lower respiration rates, higher vitamin C content, and better sensory scores than the uncoated samples after 13 days. It was reported that the beef slices sprayed with bio-composite hydrosols prepared of hydroxypropylmethyl cellulose, chitosan, lysozyme, and nano colloidal silver, inhibited microorganisms about 2.5 Log CFU/g compared to the control sample after 4 weeks of storage at 4 °C [23]. The edible antimicrobial coating containing AgNPs was also tested in vacuum packaged sausages and was able to inhibit lactic acid bacteria for 30 days at 10 °C, thus significantly increasing the shelf-life of the sausages [24]. Incoronato et al. [21] revealed that the silver-montmorillonite embedded agar coating markedly increased the shelf-life of Fior di Latte cheese due to the ability of silver cations to control microbial proliferation without affecting the functional dairy microbiota and the sensory characteristics of the product. Similarly, another study showed that AgNPs loaded in sodium alginate coating strongly controlled the microbial growth of *Pseudomonas* spp. and enterobacteria in Fior di Latte cheese, and the active coating with modified atmosphere packaging could prolong the cheese shelf-life [22]. However, few studies have applied eco-friendly produced silver-containing biopolymer films to fresh beef packaging to maintain storage quality and food safety, to the best of our knowledge.

Therefore, this work investigated the changes in microbiological and physicochemical properties of fresh beef loin packaged with the agar/AgNP composite films using conventional air-sealed packaging during storage at refrigerated temperature.

## 2. Materials and Methods

### 2.1. Materials

Food grade agar was obtained from Fine Agar Co., Ltd. (Damyang, Jeonnam, Korea). Other analytical grade chemicals, including glycerol, AgNO_3_, NaCl, and sodium citrate, were procured from Junsei Chem. Co. (Tokyo, Japan).

### 2.2. Preparation of Beef Sample

A fresh (6 days after postmortem) boneless beef loin was purchased from a local butcher, and the samples were sliced into ca. 1.0 cm thick cuts in the shape of a steak. Beef slices packaged under vacuum were transported to the laboratory and stored at 5 °C before testing. The fat content of sliced beef was 16.2 ± 2.8%, as determined by solvent extraction [26].

### 2.3. Preparation of Agar-Silver Composite Films

The silver nanoparticles (AgNPs) were prepared by reducing AgNO_3_ using the environmentally friendly method [27]. First, a varied amount (0, 1, and 2 mL) of the stock solution containing Ag of 0.04 g/mL was dissolved in 150 mL distilled water with boiling, and 2 mL of 1% trisodium citrate solution was added, and then boiled for one hour to reduce AgNO_3_. The solution turned greenish-yellow, indicating that AgNPs were formed. Then, agar (4 g) was dissolved into the silver sol containing glycerol (1.2 g) as a plasticizer with vigorous mixing for 30 min at 95 °C, and cast uniformly onto a leveled Teflon film-coated glass plate, dried for 48 h at room temperature to form agar/AgNP composite films [27]. For comparison, neat agar films were prepared following the same procedure without AgNPs. The dried films were peeled off the casting surface and conditioned at 25 °C, 50% RH at least two days before further testing. Characterization of the prepared agar/AgNP composite films has already been performed in terms of their optical (color and transparency), structural (XRD, FE-TEM, FE-SEM, and AFM), chemical (EDS and FT-IR), mechanical (tensile strength, elongation at break, and elastic modulus), barrier (water vapor permeability and water contact angle), thermal (TGA and DTG), and antimicrobial (viable cell count) properties in our previous study [27,28]. The prepared films were designated as agar, agar/AgNP^1%^, and agar/AgNP^2%^ films, respectively, according to the AgNP concentration.

### 2.4. Pathogenic Bacteria and Preparation of Inocula

The bacterial strains of *L. monocytogenes* (ATCC-19111) and *E. coli* O157:H7 (ATCC-43895) as representative Gram-positive and Gram-negative bacteria were obtained from the microbial culture collection at the Korea Food Research Institute. Authorized selective media were used for the isolation and cultivation: sorbitol MacConkey agar (Difco, Detroit, MI, USA) for *E. coli* O157:H7 and Oxford Listeria selective agar with 1 mL/100 mL supplement (Merck, Darmstadt, Germany) for *L. monocytogenes*. Both the microorganisms were spread on the corresponding media and incubated at 37 °C for 48 h.

### 2.5. Cultures and Cell Cocktails 

The preparation of the cell cocktails followed the method of Lee et al. [29]. At first, each strain was cultured in tryptic soy broth (Difco) at 37 °C for 16 h. The bacterial cultivation was repeated two times consecutively, and the resulting cultures were used as the mother cultures. Next, each mother culture was transferred individually into an appropriate broth and cultured at 30 °C for *L. monocytogenes* and 37 °C for *E. coli* O157:H7 until the late log phase. These strains were washed twice by centrifugation (PK121R, Thermo Scientific, Leicestershire, UK) at 7000 rpm for 5 min (4 °C) with a 0.85% sterile NaCl solution. Cell pellets were re-suspended in 30 mL of 0.85% sterile NaCl solution, with the final cell concentration being approximately 10^8^–10^9^ CFU/mL. Two pathogenic cultures were mixed at the same proportion to make the inoculation cocktails (about 10^5^–10^6^ CFU/mL). These culture cocktails were used in the subsequent experiments.

### 2.6. Inoculated Packaging Test

Two pathogenic bacteria were inoculated onto sliced beef, according to Koseki et al. [30]. First, beef samples were cut into 5 × 7 × 1 cm^3^ (width × length × thickness) sections which weighed about 50 g. A given amount (0.5 mL) of the cell cocktails was spotted onto the surface of sliced beef (ca. 50 g) at a level of 10^3^–10^4^ CFU/g, and then the agar composite film (11 × 11 cm^2^) was attached to both surfaces of each inoculated beef piece. Finally, the samples were hermetically packed in Ny/PE film bags (15 × 17 cm^2^) with an electric impulse sealer (TH-300, Tower Industry Co., Seoul, Korea) to avoid excess void volumes, stored at 5 °C and 85–90% RH for 15 days, and periodically analyzed for microbial population and physicochemical properties.

### 2.7. Microbial Analysis

The inoculated beef sample of ca. 50 g was mixed with 100 mL of 0.85% sterile NaCl solution and then homogenized with a stomacher (Bagmixer^®^ 400, Interscience, Bretèche, France) for 1 min. After homogenization, aliquots (1 mL) of the samples were serially diluted in 9 mL of 0.1% sterile peptone water, and 0.1 mL of the samples or diluents were surface plated onto each selective agar for pathogenic bacteria. The selective agar media were incubated at 37 °C for 24 to 48 h, and the viable cell colonies were counted. For detection of other microorganisms, 1 mL of the samples or diluents was plated in PCA (Difco) for mesophilic aerobes, MRS agar (Difco) for lactic acid bacteria, and Chromocult agar (Merck) for coliforms. These plates were incubated at 37 °C for up to 3 days, and then the viable cell colonies were counted. All the microbial counts were represented as colony forming units (CFU) per gram of samples.

### 2.8. Color

The surface color of beef samples was measured using a colorimeter (CR-400, Konica-Minolta, Tokyo, Japan). Before measurements, the instrument was calibrated on the CIE-LAB color space system with a standard white plate (L* = 97.79, a* = −0.11, b* = 2.69). Five readings at least were made separately from both surface sides of each sample, and the mean values were recorded to determine the color coordinates L* (lightness), a* (greenness/redness), and b* (blueness/yellowness).

### 2.9. TBARS Assay

Lipid oxidation of beef samples was measured by the 2-thiobarbituric acid-reactive substances (TBARS) assay as described by John et al. [31]. Duplicate beef samples (5 g) were mixed with 25 mL of a stock solution containing 0.375% TBA (Sigma Chem. Co., St. Louis, MO, USA), 15% trichloroacetic acid (Wako Pure Chem., Osaka, Japan), and 0.25 N HCl (Showa Chem., Tokyo, Japan). The mixture was homogenized for 2 min using an Ultra-Turrax tissue homogenizer (T10 digital, IKA, Staufen, German), heated for 10 min in a boiling water bath (100 °C) to develop a pink color, cooled in tap water, and then centrifuged (PK121R, Thermo Scientific, Leicestershire, UK) at 5500 rpm for 25 min. The absorbance of the supernatant was measured spectrophotometrically (V-550, Jasco, Tokyo, Japan) at 532 nm against a blank containing all the reagents minus the meat. TBA reacts to give multiple biomolecular breakdown products that have undergone free radical attack to form TBA reactive substances (TBARS), malonaldehyde (MDA) or an MDA-like derivative [32]. The TBARS contents were calculated using an extinction coefficient of 1.56 × 10^5^/M·cm, multiplying the absorbance values by 2.77 [31], and were expressed as mg of MDA equivalents per kg of beef sample.

### 2.10. Statistical Analysis 

All of the experiments were carried out independently in duplicate, and two analyses per replication were done. The results are presented as the mean and standard deviation of multiple measurements (n ≥ 4 for microbial tests and TBARS, n ≥ 20 for color). Significant differences in experimental data among packaging treatments were analyzed using the GLM procedure (SAS Institute Inc., Cary, NC, USA) at *p* < 0.05 with mean separation by LSD and Duncan’s multiple range test.

## 3. Results and Discussions

### 3.1. Packaging Effect on Microbial Population

**Figure 1:**
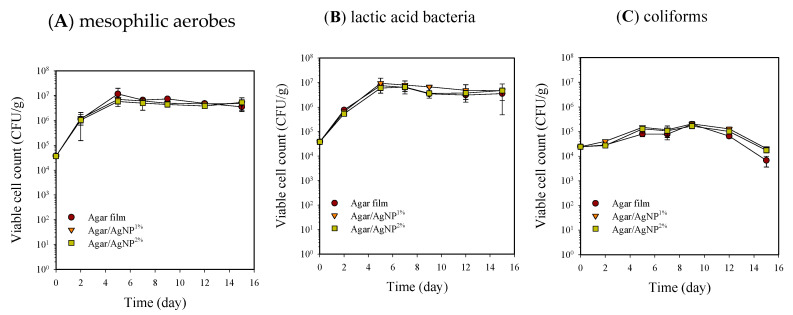
Changes in viable cell counts of mesophilic aerobes (**A**), lactic acid bacteria (**B**), and coliforms (**C**) in beef samples packaged with agar/AgNP composite films. Mesophilic aerobes: LSD_time_ = 0.12 Log (CFU/g), LSD_treatment_ = 0.08 Log (CFU/g). Lactic acid bacteria: LSD_time_ = 0.15 Log (CFU/g), LSD_treatment_ = 0.10 Log (CFU/g). Coliforms: LSD_time_ = 0.09 Log (CFU/g), LSD_treatment_ = 0.06 Log (CFU/g).

**Figure 2:**
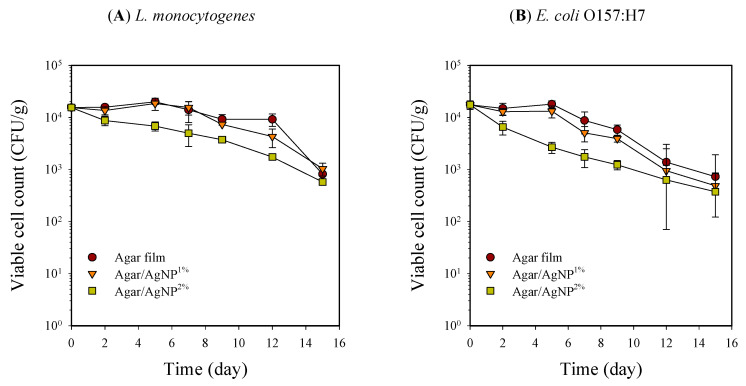
Changes in viable cell counts of *L. monocytogenes* (**A**) and *E. coli* O157:H7 (**B**) in beef samples packaged with agar/AgNP composite films. *L. monocytogenes*: LSD_time_ = 0.09 Log (CFU/g), LSD_treatment_ = 0.06 Log (CFU/g). *E. coli* O157:H7: LSD_time_ = 0.14 Log (CFU/g), LSD_treatment_ = 0.09 Log (CFU/g).

Microbial populations of beef samples packaged with the agar composite films containing 0, 1, and 2% AgNPs during storage at 5 °C are shown in Figure 1 and Figure 2. All the packaging treatments did not achieve a bactericidal or bacteriostatic effect for mesophilic aerobes and lactic acid bacteria, resulting in an increase of approximately 2 Log cycles after 15 days of storage. Previously confirmed potent antibacterial activity of the agar composite films with 1–2% AgNPs could not be observed against the natural beef microorganisms tested in the present work [27]. Such a result is not reportedly unusual because the inactivation of silver is favored by the presence of proteins and other biomolecules [11]. However, in coliform bacteria, viable cell counts increased by 1 Log CFU/g until nine days of storage and then decreased by more than 1 Log cycle. Thus, the reduction of coliform bacteria was presumably associated with the growth of lactic acid bacteria on beef samples.

Inoculated pathogenic bacteria, *L. monocytogenes* and *E. coli* O157:H7, on beef samples could not properly proliferate, and the viable cell counts decreased with time, depending on packaging treatment. Initial inoculants of *L. monocytogenes* and *E. coli* O157:H7 onto beef samples were at the level of 1.8 ± 0.3 × 10^4^ and 1.6 ± 0.2 × 10^4^ CFU/g, respectively. Each microbial population reduced noticeably to 7.3 ± 0.1 × 10^2^ and 8.1 ± 0.5 × 10^2^ CFU/g after 15 days of storage at 5 °C. Particularly, beef samples packaged with 2% silver-incorporated agar film showed significantly (*p* < 0.05) lower viable cell counts than those with neat agar film over the entire storage period (Figure 2). The difference in antimicrobial activity against native microorganisms and inoculated pathogens of the agar/AgNP composite film can be attributed to differences in initial load, dispersion of test bacteria on the surface of beef samples, and differences in sensitivity to AgNP [33,34]. Similarly, about one log cycle reduction of *Pseudomonas putida* inoculated as a psychrotrophic spoilage bacterium was found on raw beef, pork, and turkey cuts covered with silver ion impregnated wrapping paper compared to those with regular butcher paper during storage at 10 °C for four days [10]. Martínez-Abad et al. [11] also reported similar results on the pathogen experiment in which chicken breasts were inoculated with *L. monocytogenes* and packed with EVOH film containing 1% and 10% silver ions. Samples with a high amount of silver (10%) only exhibited microbial count values up to 1 Log CFU/g lower than the control without silver after 72 h of incubation at 12 °C.

Silver damages bacteria by unspecific binding to membrane and respiratory enzymes [35,36]. Moreover, silver nanoparticles may accumulate in the bacterial cytoplasmic membrane, causing a remarkable increase in membrane permeability and leading to cell death [37]. This antimicrobial action of silver or silver ions exhibits effectiveness for Gram-positive and Gram-negative bacteria from the previous studies, using a silver solution, silver nanoparticles, and silver-casting film [11,18,27,28,38]. In this study, silver released from the agar film with 2% AgNPs showed a significant bactericidal effect for *L. monocytogenes* and *E. coli* O157:H7 inoculated on beef samples.

Overall reduction of the inoculated pathogens on beef samples packaged in the agar films with or without AgNPs was probably due to the proliferation of lactic acid bacteria during storage. The addition of certain strains of lactic acid bacteria to ground beef reportedly inhibited the growth of pathogenic bacteria such as *Salmonella*, *E. coli*, and *Staphylococcus* at refrigeration temperatures [6,39,40]. Reduction of viable cell counts by 2–3 Log cycles for *Salmonella* and *E. coli* O157:H7 could be achieved with lactic acid bacteria of about 10^7^ CFU/g after storage of 5 days at 5 °C [39].

### 3.2. Packaging Effect on Surface Color

**Figure 3:**
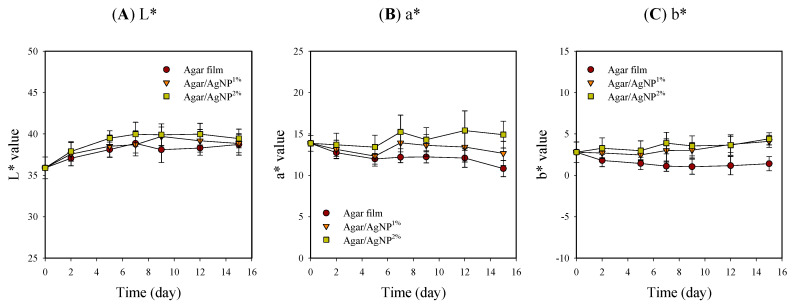
Changes in L* (**A**), a* (**B**), and b* (**C**) values of beef samples packaged with agar/AgNP composite films. L* value: LSD_time_ = 0.42, LSD_treatment_ = 0.27. a* value: LSD_time_ = 0.48, LSD_treatment_ = 0.30. b* value: LSD_time_ = 0.34, LSD_treatment_ = 0.22.

Appearance and color values, including L*, a*, and b*, of beef samples with packaging treatment, are shown in Table 1 and Figure 3. The surface color of meat is one of the critical quality factors in customer choice because the color is an indicator of freshness [41]. Packaging treatment had no significant effects on L* values that gradually increased and leveled off in the ranges of 35.9–40.0 during storage. However, in the case of a* and b*, initial values were kept relatively constant in the beef samples with the silver-incorporated agar films over time. In contrast, the color values slightly decreased initially from 13.9 ± 1.0 and 2.8 ± 1.2 down to 10.8 ± 1.0 and 1.1 ± 0.9, respectively, in the control. The control samples became slightly brownish (i.e., reduced redness) with storage time. Such a color change in the control group might be presumably attributed to lipid oxidation and metmyoglobin formation [4,9,42]. Murphy et al. [43] also revealed that the decrease in a* values coincided with increased lipid oxidation in beef steak after 14 days of storage at 4 °C. Present results suggest that the agar/AgNP composite films could act as an oxygen gas barrier preventing beef from direct contact with oxygen. The oxygen permeability of neat agar film was evaluated as 2.11 mL∙mm/m^2^∙day∙atm to be remarkably reduced by incorporating Ag-Cu nanoparticles and is comparable to that of a commercial barrier polymer [44]. The hydrocolloid-based biopolymer films reportedly have excellent oxygen barrier properties under dry conditions, whereas they are very poor moisture barriers [20]. Thus, oxygen barrier properties under wet conditions become weaker due to the water vapor absorption, leading to a loss of network by swelling and plasticization. However, composite materials formed through the polymer blending process generally exhibit the complementary advantages of each component and minimize their disadvantages [18]. Consequently, it is notable that the agar composite films have exerted a positive effect, to some extent, on keeping the characteristic red color of fresh beef samples during cold storage.

### 3.3. Packaging Effect on Rancidity

**Figure 4:**
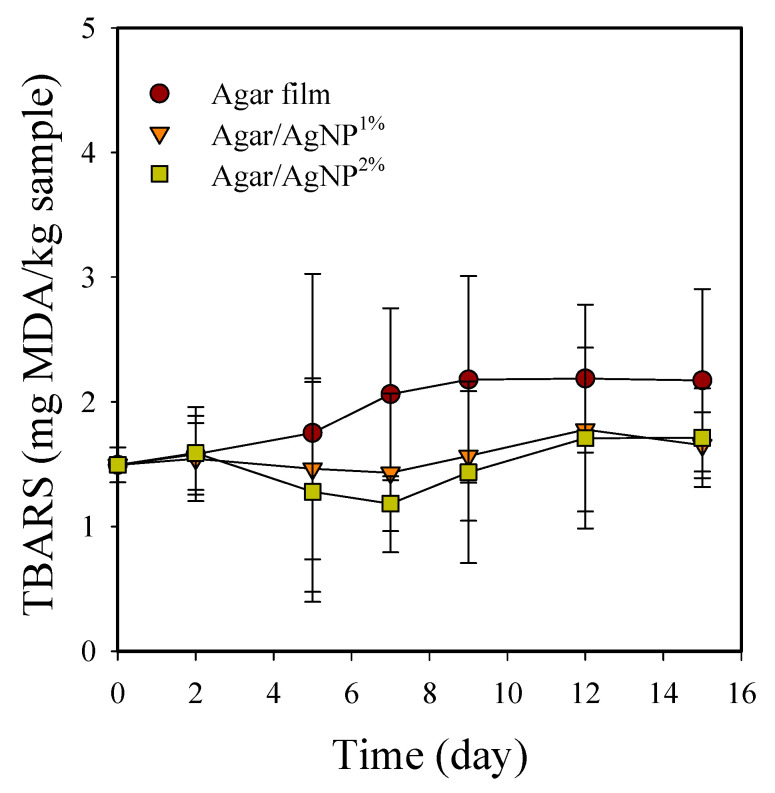
Changes in TBARS of beef samples packaged with agar/AgNP composite films. TBARS: LSD_time_ = 0.59, LSD_treatment_ = 0.39.

TBARS of packaged beef samples increased significantly (*p* < 0.05) during storage at 5 °C (Figure 4). TBARS value was initially 1.5 ± 0.1 mg MDA/kg and gradually increased to 2.2 ± 0.6 mg MDA/kg in the control samples after 15 days. TBARS is well known to correlate with off-flavor development in chill-stored beef [45]. In general, lipid oxidation results in odor and flavor degradation as unsaturated fatty acids react with molecular oxygen through free radical transfer reactions to form fatty acyl hydroperoxides or peroxides, which cause off-flavors [5]. Lipid oxidation is also reportedly associated with metmyoglobin formation and meat discoloration [46]. Campo et al. [47] have previously demonstrated that off-flavors such as rancidity in beef can be detected by humans when TBARS values are greater than or equal to 2.0 mg MDA/kg meat. Only the control group showed TBARS values above 2.0 mg MDA/kg beef after seven days in our present study. However, in the beef samples packaged with the silver-incorporated agar films, TBARS values remained below the acceptable threshold of 2.0 mg MDA/kg beef over the storage period. The results indicate that the agar/AgNP composite films partially working as an antioxidant could retard oxidative rancidity in the stored meat. Indeed, AgNPs stabilized with cellulosic polymers have shown the notable antioxidant activity measured as the DPPH scavenging percentage at the level of approximately 30–40% of ascorbic acid used as a positive standard [48].

The practical use of AgNP-containing films in food packaging applications may raise concerns from a food safety point of view. One of the major concerns about using such packaging materials is insufficient knowledge about the safety and toxicity of AgNPs [20]. Although AgNPs may kill human cells, as shown in vitro [49], AgNPs-impregnated cellulose nanofibril composite films have shown no significant cytotoxicity to human epithelial cell or colon cell lines [19,50]. There is little information about the migration or release of AgNPs from biopolymers to food surfaces and their possible risks to human health [51]. In general, it has been reported that nanoparticles embedded in a host polymer matrix are not likely to migrate into food when fully embedded in the polymer, and the contact surface is not altered by mechanical surface stress during application [52]. However, the antimicrobial function of the agar/AgNP composite films is related to the release of silver ions or interactions between AgNPs and the microorganisms by direct contact. The migration process may depend little on the diffusion-controlled mass transfer of AgNPs within the polymer to the surface but is mainly induced by the oxidative dissolution of silver from the surface near the AgNPs [52]. In this regard, EU safety regulations have defined an upper limit for Ag migration in packaging that the presence of silver ions in the food matrix should not exceed 0.05 mg/kg of food [53]. Meanwhile, in this study, a green synthesis method was applied to prepare an agar/AgNP complex using citrate as a reducing agent and agar as a capping agent to overcome the toxicological behavior of silver and its compounds. Eco-friendly green chemistry methodologies to obtain metal nanoparticles and biopolymer-based composites are well known to possibly eliminate toxic chemicals and allow bio-nanocomposites in food packaging and various biological applications [19,34].

## 4. Conclusions

Sealed packaging with the agar/AgNP composite films showed a bactericidal effect on the foodborne pathogenic bacteria, including *L. monocytogenes* and *E. coli* O157:H7, of beef loins. However, no significant antimicrobial effect was found against the mesophilic aerobes, lactic acid bacteria, and coliform bacteria. The composite films could also function as an oxygen gas barrier or an antioxidant to keep the characteristic red color and inhibit lipid oxidation of stored raw beef cuts. Further research is needed to improve the effectiveness of sealed packaging with the agar/AgNP composite films. Combining antimicrobial packaging with modified atmosphere conditions can provide a much more successful way to maintain the quality attributes of fresh red meats and secure food safety during storage. For the practical use of AgNP-containing films in food packaging applications, however, assessing the real amount of silver in contact with food is required to verify the fulfillment of the EU and other regulations. In this aspect, more research should be carried out on the migration of AgNPs from the packaging to the food matrix and their cytotoxic effects on human health.

## Figures and Tables

**Table 1:** The appearance of beef samples packaged with agar/AgNP composite films.

Initial	Storage Time (day)	Agar Film	Agar/AgNP^1%^	Agar/AgNP^2%^
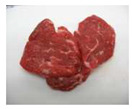	0	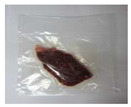	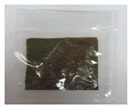	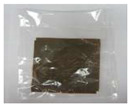
	5	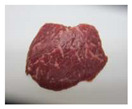	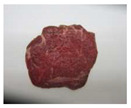	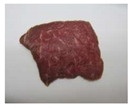
	9	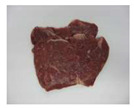	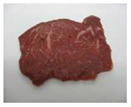	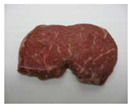
	15	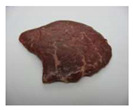	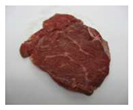	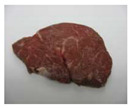

## Data Availability

Not applicable.
